# Development and Validation of a Simple High Performance Liquid Chromatography/UV Method for Simultaneous Determination of Urinary Uric Acid, Hypoxanthine, and Creatinine in Human Urine

**DOI:** 10.1155/2018/1647923

**Published:** 2018-05-15

**Authors:** Nimanthi Wijemanne, Preethi Soysa, Sulochana Wijesundara, Hemamali Perera

**Affiliations:** ^1^Department Biochemistry and Molecular Biology, Faculty of Medicine, University of Colombo, Colombo, Sri Lanka; ^2^Department of Psychological Medicine, Faculty of Medicine, University of Colombo, Colombo, Sri Lanka

## Abstract

Uric acid and hypoxanthine are produced in the catabolism of purine. Abnormal urinary levels of these products are associated with many diseases and therefore it is necessary to have a simple and rapid method to detect them. Hence, we report a simple reverse phase high performance liquid chromatography (HPLC/UV) technique, developed and validated for simultaneous analysis of uric acid, hypoxanthine, and creatinine in human urine. Urine was diluted appropriately and eluted with C-18 column 100 mm × 4.6 mm with a C-18 precolumn 25 mm × 4.6 mm in series. Potassium phosphate buffer (20 mM, pH 7.25) at a flow rate of 0.40 mL/min was employed as the solvent and peaks were detected at 235 nm. Tyrosine was used as the internal standard. The experimental conditions offered a good separation of analytes without interference of endogenous substances. The calibration curves were linear for all test compounds with a regression coefficient, *r*^2^ > 0.99. Uric acid, creatinine, tyrosine, and hypoxanthine were eluted at 5.2, 6.1, 7.2, and 8.3 min, respectively. Intraday and interday variability were less than 4.6% for all the analytes investigated and the recovery ranged from 98 to 102%. The proposed HPLC procedure is a simple, rapid, and low cost method with high accuracy with minimum use of organic solvents. This method was successfully applied for the determination of creatinine, hypoxanthine, and uric acid in human urine.

## 1. Introduction

In purine catabolism, xanthine oxidase oxidizes hypoxanthine to xanthine and then xanthine to uric acid. The balance between the uric acid synthesis and degradation maintains uric acid levels in serum. The abnormal levels of uric acid in serum and urine are associated with defects in enzymes in purine metabolism [1, 2] and are reported in cardiovascular diseases, gouty arthritis, renal disease, type 2 diabetes, obesity, and hypertension [2–8].

The total daily excretion of creatinine at steady state is considered to be proportional to the total-body creatinine content and total-body skeletal muscle mass [9]. Therefore the levels of creatinine have been used to standardize the concentration of urinary constituents to improve the accuracy of the analysis [10–12]. In most studies colorimetric methods are used to determine urinary creatinine levels [13]. Employing a simultaneous analysis of purine metabolites together with creatinine is a better approach to improve the accuracy of the measurements and also it reduces the time, labour, and cost per sample. HPLC methods for simultaneous quantification of creatinine, uric acid, and hypoxanthine have been reported. Analysis of creatinine and purine derivatives in ruminant urine has been described using HPLC with gradient or isocratic solvent systems [14–16]. HPLC analysis of uric acid and hypoxanthine in human urine has also been reported [13]. A more complex method with liquid chromatographic system equipped with automatic column-switching valves has been used to analyse creatinine, hypoxanthine, and uric acid [17].

The present method employs a further improvement of our previous study [18]. An internal standard was used to improve the accuracy in the quantification of analytes. The method developed was applied to determine urinary creatinine, uric acid, and hypoxanthine levels in children with autism.

## 2. Instrumentation

HPLC was performed with Shimadzu LC10AS solvent delivery system equipped with Shimadzu SPD-10AVP UV visible detector, DGU-14A degaser (Shimadzu, Kyoto, Japan), chromolith RP-18e 25-4.6 mm column (the precolumn) coupled in series with chromolith flash, and RP 18 e 100-4.6 mm column. Microcentrifugation of samples was carried out in Biofuge D-37520 (Heraeus instruments) centrifuge. Samples were injected with a syringe loading injector fitted with 20 *μ*L loop.

## 3. Reagents

Acetonitrile (HPLC grade), sodium acetate, and tyrosine were purchased from BDH (BDH Chemicals Limited, Poole, England). Dipotassium hydrogen orthophosphate, potassium dihydrogen phosphate, creatinine, uric acid, hypoxanthine, and sodium hydroxide were purchased from Riedel-de Haen AG, Seelze, Hannover. Deionized water to prepare the standards and the mobile system were obtained from Milli Q50 water purification system (Millipore, USA).

## 4. Collection of Urine

Six samples of human urine were analyzed for creatinine, uric acid, and hypoxanthine as an application of the method. Urine was collected to sterile plastic bottles. The total volume was measured. Aliquots (2 mL in duplicate and 10 mL) were centrifuged at 2500 rpm for 5 min and the supernatant was transferred to autoclaved sterile tubes. The lids were covered with parafilm, and samples were stored at −20°C until analyzed. The sample analysis was done within 24 hours of collection.

## 5. Preparation of Mobile Phase

The mobile phase, potassium phosphate buffer (20 mM, pH 7.25), was prepared by mixing K_2_HPO_4_ (240 mL; 20 mM) with KH_2_PO_4_ (60 mL; 20 mM).

## 6. Preparation of Standards

The stock standards were prepared at a concentration of 500 *μ*g/mL for each analyte, uric acid, creatinine, and hypoxanthine in 100 mL volumetric flasks with deionized water or urine. Few drops of NaOH (0.5 M) were added to dissolve hypoxanthine and uric acid. The standards were stored at −20°C. Calibration standards consisting of uric acid, creatinine, and hypoxanthine were prepared by appropriate dilutions of the stock standards with deionized water. Tyrosine (250 *μ*g/mL) dissolved in acetonitrile (100%) was used as the internal standard.

## 7. Sample Preparation for HPLC Analysis

Chromatographic separation was achieved by coupling RP-18e 25-4.6 mm column (precolumn) with RP 18e 100-4.6 mm column. Internal standard, tyrosine (100 *μ*L), was added to each standard mixture or urine (100 *μ*L) and vortex mixed for 30 seconds followed by centrifugation (5 min at 3000 rpm); the supernatant (20 *μ*L) was injected.

## 8. Chromatographic Conditions

The optimum separation was achieved by potassium phosphate buffer (20 mM, pH 7.25) at a flow rate of 0.40 mL/min. Effluents were monitored at different UV wave lengths and optimum sensitivity was detected at 235 nm for purine metabolites, creatinine, and the internal standard. Interference of tyrosine was not observed with retention times of analytes or endogenous substances present in urine.

The mobile phase was pumped for 30 min time period through the column before the commencement of analysis. Column was flushed with degassed, deionized water for 20 min and then with 8% acetonitrile for 20 min after analysis of samples. The columns were left with 8% acetonitrile until the next usage to protect them from microbes.

## 9. Method Validation

The method was validated according to the US-FDA Bioanalytical Method Validation Guidance to evaluate selectivity, linearity, lower limit of quantification (LLOQ), precision and accuracy, recovery, and stability.

Quality control (QC) samples were prepared at concentrations of 12.5, 25.0, and 75.0 *μ*g/mL for each analyte using deionized water and stored at −20°C. QC samples were used for method validation and sample analysis to ensure the quality of data.

Limit of Detection (LOD) and Limit of Quantification (LOQ) were analyzed based on the standard deviation (SD) of the response and the slope. Five calibration curves were constructed for each analyte in the concentration range of 3.13–25.0 *μ*g/mL. The SD obtained at each concentration was plotted against the concentration. The intercept at zero concentration (SD_0_) obtained by extrapolation of the curve was used to evaluate the LOD and LOQ. The LOD was calculated as the 3*∗*SD_0_ and LOQ was 10*∗*SD_0_ [19]. The lower limit of quantitation (LLOQ) was determined from the lowest concentration of the standard curve with an acceptable accuracy and precision in analysis.

The standards were diluted in water to obtain different concentrations of uric acid (3.12–200 *μ*g/mL), creatinine (3.12–100 *μ*g/mL), and hypoxanthine (6.25–200 *μ*g/mL) and used to determine the linearity of the calibration curve. Matrix effects were also studied after diluting the standards with urine.

The calibration curve was obtained with concentration against corresponding peak area ratio. The slope and intercept of the calibration curves were calculated through least squares linear regression analysis using Microsoft Excel 2013. The slope and the intercept of the calibration curves were obtained over a period of 6 weeks using eight independent series of the calibration standards prepared as described above.

The samples were stored at −20°C. Three concentrations (12.5, 25, and 75 *μ*g/mL) with three freeze thaw cycles over six weeks were evaluated for stability. The observed values were compared with the freshly made relevant standards.

## 10. Application of the Proposed Method

Urine samples of six autism patients were analyzed as an application of the developed method. The dilutions of urine were prepared by observing the initial HPLC chromatogram in quantification of purine metabolites and creatinine and 20x and 40x dilutions were made accordingly depending on their concentrations. Dilutions at 4x dilutions were made in the analysis of hypoxanthine in urine. Calibration curves were constructed simultaneously in sample analysis to calculate the urinary levels of uric acid creatinine and hypoxanthine.

## 11. Results and Discussion

### 11.1. Optimization of Chromatographic Separation

Different approaches have been published for simultaneous determination of uric acid, hypoxanthine, and creatinine [12–18, 20]. The method described in the present study employs a precolumn in series for better resolution of analytes and the internal standard at a shorter period of run time for a single injection.

The mobile phase composition was optimized to separate all the analytes as well as the internal standard without interfering coeluting endogenous substance following injection of blank urine samples. Several modifications of the composition of the mobile phase were carried out to find out a best resolution. The detection UV wave length for effluents was also studied to obtain the maximum sensitivity to quantify the analytes simultaneously. Effects of pH (3 to 7.25), flow rate, and the molarity of the running buffer were optimized using standards and human urine samples to improve the precision and accuracy. Uric acid (pKa value 5.4) showed fluctuations of retention time with the pH compared to creatinine and hypoxanthine. Optimum chromatographic conditions for the simultaneous analysis of uric acid, creatinine, and hypoxanthine were obtained with potassium phosphate buffer (20 mM, pH 7.25) at a flow rate of 0.4 mL/min with a detection wave length of 235 nm. All the experiments were carried out at room temperature.

The column was washed with deionized water daily to avoid the risk of precipitating salts in the column and kept in 8% acetonitrile to prevent fungal and bacterial attacks due to the usage of buffers. The daily washing and storing process with acetonitrile also prevented the aggregation of the bonded hydrocarbon chains which may occur due to the usage of aqueous mobile phases [20].

## 12. Validation of the HPLC Assay

The developed HPLC assay was validated for linearity, specificity, stability, accuracy, precision, and repeatability. The limit of detection (LOD) and limit of quantification (LOQ) were also determined.

## 13. Selectivity and Specificity

**Figure 1:**
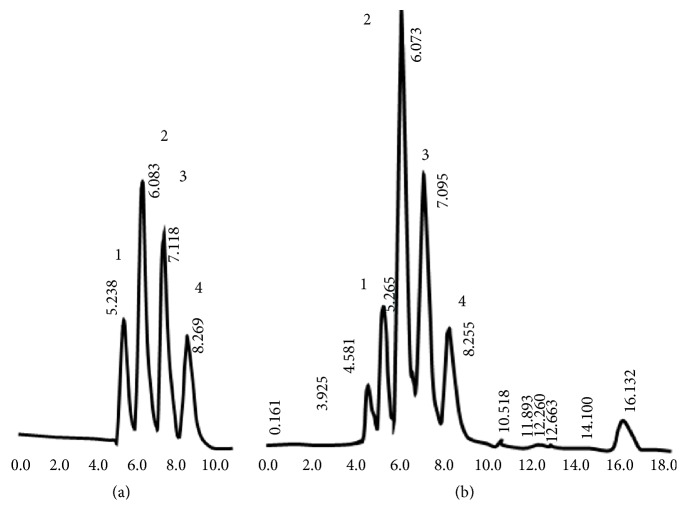
HPLC chromatograms of (a) standards of uric acid (75 *μ*g/mL), creatinine (75 *μ*g/mL), internal standard, tyrosine (125 *μ*g/mL), and hypoxanthine (75 *μ*g/mL). 1, uric acid (5.2 min); 2, creatinine (6.0 min); 3, tyrosine the internal standard (7.1 min); 4, hypoxanthine (8.2 min). (b) HPLC chromatogram of human urine.

The samples were initially run for 45 mins to screen any endogenous substances, which might affect the next sample injection. Chromatograms of blank urine samples were examined and the method was optimized to avoid the interference from the analyte peaks with endogenous peaks of the urine and the internal standard. No endogenous interfering peaks were observed in the individual blank sample at the retention time (RT) of the three analytes and the internal standard. Although the peaks appeared broad due to passage through two columns, excellent chromatographic separation was obtained with RT of 5.2, 6.0, 7.1, and 8.2 mins for uric acid, creatinine, Internal standard (tyrosine), and hypoxanthine, respectively.  Figure 1 illustrates representative chromatograms of a mixture of standards in water and urine spiked with the internal standard under the described chromatographic conditions.

## 14. Linearity

**Figure 2:**
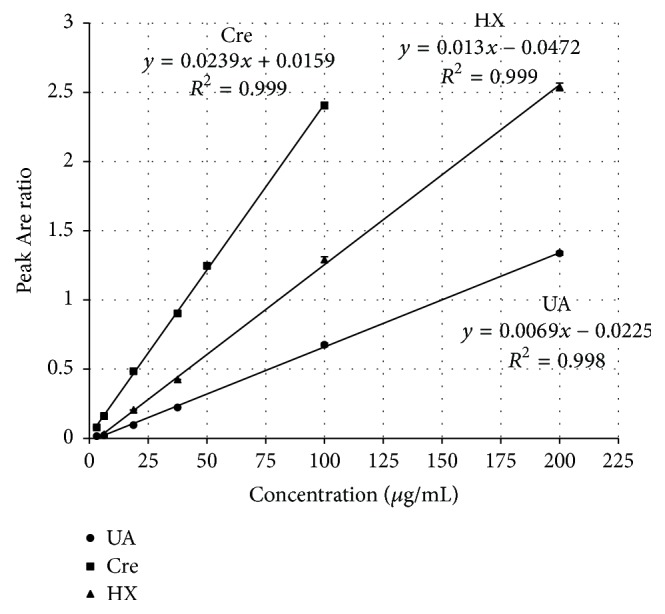
Calibration curves obtained for uric acid (UA; 3.12–200 *μ*g/mL), creatinine (Cre; 3.12–100 *μ*g/mL), and hypoxanthine (HX; 6.25–200 *μ*g/mL) (*n* = 5).

Intraday (*n* = 5) was established by constructing calibration curves for standards of uric acid (3.12–200 *μ*g/mL), hypoxanthine (6.25–200 *μ*g/mL), and creatinine (3.12–100 *μ*g/mL) in water. The ratio of the peak area of the standards to that of the internal standard against the concentration in aqueous solutions was linear (Figure 2) and the correlation coefficient (*r*^2^) values were >0.99 (Table 1).

## 15. Stability of Samples

All three analytes were stable at −20°C storage and the percentage deviation of concentrations from the reference standards was less than 12% at high, medium, and low concentrations. The gradient of the calibration curve also remained stable over the concentration range investigated, during six weeks, when stored at −20°C. The values were compatible with the slope from the freshly made standards suggesting short and long term stability with minimal intraday and interday variability at measured levels over six weeks.

Accuracy was determined by the analysis of recovery. Known amounts of analytes were spiked with water at three concentration levels of each analyte (12.5, 25, and 75 *μ*g/mL). The experiment was performed in triplicate and the percentage recovery was determined using the calculated value, obtained from the calibration curve, to the true value (Table 2).

(1) $$\begin{array}{c} \%\;\;Recovery=\left(\;\frac{calculated\;\;amount}{theoretical\;\;amount}\;\right)\times 100\%. \end{array}$$

The recovery for the three analytes at 12.5, 25, and 75 *μ*g/mL was obtained between 97 and 102% indicating the accuracy of the developed method.

## 16. Repeatability

Coefficient of variation (CV%) for the repeated injections (*n* = 3) at three quality control levels (12.5, 25, and 75 *μ*g/mL) was analyzed (Table 2). The values (0.15–0.95%) were within the accepted range (15%) indicating the repeatability of the present validated HPLC/UV method.

## 17. Precision

Intraday (*n* = 5) and interday (*n* = 8) variations were evaluated for precision, by analyzing the quality control samples of the three analytes over six weeks and CV% were calculated (Table 2).

The CV% for the three analytes obtained were lower than the recommended value (15%) proving the intermediate precision of the developed HPLC/UV method.

## 18. Limit of Detection (LOD), Limit of Quantification (LOQ), and Lower Limit of Detection (LLOD)

LOD, LOQ, and LLOQ are given in the Table 3.

## 19. Patient Samples

Urine samples from six individuals were analyzed (Table 4).

## 20. Conclusion

The present study describes a simple, less expensive isocratic method for the simultaneous analysis of uric acid, creatinine, and hypoxanthine. The method can be employed for the analysis of purine metabolism defects using human urine samples with normalization with creatinine.

## Figures and Tables

**Table 1:** Regression equation obtained for interday and intraday variation of uric acid, creatinine, and hypoxanthine.

Experiment	Analyte	Regression equation	*r* ^2^
Intraday variation *n* = 5	Uric acid	*Y* = 0.0069*x* − 0.022	0.998
	Creatinine	*Y* = 0.0236*x* + 0.016	0.999
	Hypoxanthine	*Y* = 0.0124*x* − 0.0466	0.999

Interday variation *n* = 8	Uric acid	*Y* = 0.006*x* − 0.008	0.995
	Creatinine	*Y* = 0.0218*x* + 0.028	0.998
	Hypoxanthine	*Y* = 0.0124*x* − 0.006	0.997

*Y*, peak area ratio (peak area of the analyte to that of the internal standard); *x*, concentration of the analyte; *r*^2^, correlation coefficient.

**Table 2:** Accuracy, repeatability, and precision for uric acid, creatinine, and hypoxanthine.

	Accuracy (recovery%) *n* = 5	Repeatability CV%	Precision (CV%)	
			Intraday (*n* = 5)	Interday (*n* = 8)
Uric acid (*μ*g/mL)				
12.5	98.17 ± 0.57	0.35	0.74	4.62
25	100.12 ± 0.16	0.50	1.20	3.84
75	99.49 ± 0.13	0.15	0.53	1.64
Creatinine (*μ*g/mL)				
12.5	97.52 ± 0.38	0.95	1.88	4.40
25	101.44 ± 0.21	0.56	0.59	4.58
75	99.84 ± 0.42	0.81	0.73	1.41
Hypoxanthine (*μ*g/mL)				
12.5	100.32 ± 1.75	0.35	0.31	3.53
25	100.43 ± 1.02	0.63	0.37	3.13
75	100.40 ± 0.15	0.19	1.64	1.40

CV%, coefficient of variation; *n*, number of replications of analyte samples.

**Table 3:** Limit of quantification (LOQ), limit of detection (LOD), and lower limit of quantification (LLOQ) for uric acid, creatinine, and hypoxanthine.

Analyte	LOQ (*μ*g/mL)	LOD (*μ*g/mL)	LLOQ (*μ*g/mL)
Uric acid	0.89	0.30	3.12
Creatinine	1.05	0.35	3.12
Hypoxanthine	0.84	0.28	6.25

**Table 4:** Concentrations of uric acid (UA), hypoxanthine (HX), and creatinine (Cre) and the ratios for purine metabolite to creatinine in urine samples of six individuals.

Cre (*μ*g/mL)	UA (*μ*g/mL)	HX (*μ*g/mL)	UA/Cre	HX/Cre
1425.54	739.43	364.13	0.52	0.25
850.30	675.38	114.34	0.79	0.13
621.95	383.14	61.31	0.62	0.10
1056.72	918.89	93.95	0.87	0.09
1082.33	1123.41	347.63	1.04	0.32
1116.87	1737.85	373.85	1.56	0.33

## Data Availability

The data [HPLC chromatogram, graphics, and tables] used to support the findings of this study are included within the article. All the necessary data are included in the manuscript and data sharing is not applicable to this paper.
